# High carbon-di-oxide modified atmospheric packaging on quality of ready-to-eat minimally processed fresh-cut iceberg lettuce

**DOI:** 10.1007/s10068-021-00881-4

**Published:** 2021-03-01

**Authors:** Md. Azizul Haque, Md. Asaduzzaman, Md. Sultan Mahomud, Md. Rizvi Alam, Alin Khaliduzzaman, Shib Nath Pattadar, Raju Ahmmed

**Affiliations:** 1 Faculty of Science and Technology, Free University of Bozen-Bolzano, Piazza Università 1, 39100 Bolzano, Italy; 2 Department of Food Technology and Nutritional Science (FTNS), Mawlana Bhashani Science and Technology University (MBSTU), Tangail, 1902 Bangladesh; 3 Department of Food Engineering and Technology, Hajee Mohammad Danesh Science and Technology University, Dinajpur, 5200 Bangladesh; 4 Graduate School of Agriculture, Kyoto University, Kitashirakawa-Oiwake-cho, Sakyo-ku, Kyoto, 606-8502 Japan; 5 Department of Food Engineering and Technology, Sylhet Agricultural University, Sylhet, 3100 Bangladesh; 6 School of Environment and Natural Resources, The Ohio State University, Columbus, OH 43210 USA; 7 Department of Chemical and Food Engineering, Dhaka University of Engineering and Technology, Gazipur, 1707 Bangladesh

**Keywords:** High CO_2_, MAP, Minimal process, Fresh-cut, Iceberg lettuce

## Abstract

Fresh-cut lettuce is a very well-known salad for today's routines because it obliges minimal preparation to minimize the loss of health beneficial vitamins, minerals, antioxidants and other phytochemicals. It is a prodigious challenge to serve its consumers fresh. Quality of freshly processed lettuce under high CO_2_ modified atmosphere packaging (MAP) has been investigated as a realistic alternative technique for its preservation. Storage under high CO_2_ atmospheric treatments exhibited a significant impact in microbial development, electrolyte leakage, volatile metabolites and sensory quality of fresh-cut iceberg lettuce. This storage condition (MAP 1: 5 kPa O_2_ and 20 kPa CO_2_ balanced by N_2_ at 7 °C for 6 days) inhibited the growth of mesophilic bacteria and yeasts; delayed the enzymatic browning (cut-edges and intact surface) of fresh-cut iceberg lettuce and overall visual quality was also in acceptance limit. The development of off-odors was perceived in high CO_2_ MAP as a consequence of volatiles (ethanol and acetaldehyde) accumulation which was persisted at an inexcusable level during 6 days of storage periods.

## Introduction

Minimally processed vegetables (MPV) are partial or completely ready and are promoted as prepared to use for the consumers (Delaquis et al., 2004; Siddiqui et al., 2020). MPV is the most apposite meal in everyone´s demanding and compressed work plan. MPV requires negligible preparation and provide great varieties of vitamins, minerals, antioxidants and bioactive compounds or phytochemicals that are essential to reduce the oxidative stress in human physiology. Those are also free from synthetic additives during the preservation periods. Minimally processed fresh-cut lettuce enhances the flavor and color in industrially manufactured foods (Rico et al., 2007; Tomás-Callejas et al., 2011). Its production involves simply washing, peeling and slicing (Rico et al., 2007).

During minimal processing, cut and exposed areas cause high perishability (Watada and Qi, 1999). Fresh-cut vegetables continue to deteriorate faster than whole vegetables due to different types of biochemical reactions, enzymatic activities associated with wound and post-harvest handling, processing and preservation (Caleb et al., 2013). Besides quality, biological safety measurement (BSM) is also important. Research outcomes showed that foodborne epidemics concomitant with lettuce, salad, and melon are augmented more during the last 30 years (Garcia and Barrett, 2002; Rodríguez-Caturla et al., 2012). In order to maintain the quality of freshly processed ready to eat vegetables, proper and suitable preservation and storage techniques are not only very important but also a big challenge for this emerging sector. Research interest on the applications of modified atmosphere packaging (MAP) is enormously increasing to maintain the quality of freshly processed ready to eat. The basic mechanism of MAP is to suppress the metabolic activity of the product by reducing the O_2_ and rising the CO_2_ concentration inside the package (López-Gálvez et al., 2015). In actual preservation practice of minimally processed iceberg lettuce, low O_2_ levels in the packages trigger volatile production and off-odor development. Alternatively, high O_2_ was also investigated to extend the shelf-life of MPV, especially iceberg lettuce and it was reported that the high O_2_ is advantageous to prevent enzymatic discoloration, anaerobic fermentation reactions and microbial growth (López-Gálvez et al., 2015). But overestimated O_2_ (>25% O_2_) might be explosive (Kader and Ben-Yehoshua, 2000). High O_2_ levels may affect substrate self-consciousness of the enzyme polyphenol oxidase (PPO) and cause feedback reticence (Chua et al., 2008). Moreover, a high O_2_ atmosphere is also associated with some other physiological disorders of lettuce, i.e. ethylene production and russet spotting. Russet spotting and ethylene production has great undesirable possessions on the overall visual quality and sensation of lettuce (López-Gálvez et al., 2015). Among other alternatives, few combinations of O_2_ and CO_2_ balanced N_2_ were uninterruptedly established by choosing the packaging film with the correct penetrability (Jacxsens et al., 2001)_._ It has been reported that the MAP (3 to 5 kPa O_2_ and 5 to 10 kPa CO_2_) has a positive impact on reducing the deterioration of MPV ( Allende et al., 2002; Amanatidou et al., 2000a; Kader and Ben-Yehoshua, 2000). On the other hand, appropriate permeable films which are primarily blushed with low O_2_ and high CO_2_ provide an extended storage life of minimally processed lettuce (López-Gálvez et al., 1996) but the most significant and conspicuous problem in high CO_2_ condition is the development of off-flavor (Jacxsens et al., 1999). On the other hand, brown stain development is one of the drawbacks of preserving fresh-cut lettuce at high O_2_ MAP (López-Gálvez et al., 1996). Therefore, a comprehensive understanding of product, MAP and storage is essential for ensuring their naturalness and nutritional values of fresh-cut vegetables. It is a thought-provoking task to develop the most effective procedures and innovative technologies for maintaining their quality as well as reducing the off-odors to meet increasing consumer demand. In our previous work (López-Gálvez et al., 2015), we have focused on the effect of high oxygen treatment on the fresh-cut lettuces. Precisely this research focused on the effect of high carbon dioxide atmospheric packaging treatment during storage on different quality parameters of fresh-cut lettuce (microbial ecology, electrolyte leakage, volatiles formed during storage, and sensory quality).

## Materials and methods

### Minimal processing of sample

Samples, the iceberg lettuce (*Lactuca sativa* L.) crowns were collected from the local fresh-cut produce industry of Ghent, Belgium. All of the step-wise minimal processing was followed by López-Gálvez et al. (2015).

### Culture media

The reagents and associated items used for microbial growth experiments were obtained in standard grade. Peptone physiologic salt (PPS) solution (8.5 g/L NaCl salt, NaCl; Fluka Chemie AG, Switzerland) and 1 g/L neutralized bacteriological peptone (Oxoid, Hampshire, England), Yeast extract glucose chloramphenicol (YGC) agar (BIO-RAD, Marnes-La-Coquette, France), Plate count agar (PCA; Oxoid, Hampshire, England).

### High CO_2_ experimental design

50 g of lettuce was directly poured into individual bags after the completion of the washing and centrifugation processes. The different types of MAP were applied for experimental design. Two MAP treatments and control samples were bred and stored at 7 °C for 6 days. Different gas concentrations used were as below:

#### MAP 1

The MAP1 procedure comprised of packaging lettuce originally at around 5 kPa O_2_ and 20 kPa CO_2_ well-adjusted by N_2_, with film permeability of 400 mL O_2_/m^2^·d·atm.

#### MAP 2

The MAP 2 technique comprised of packaging lettuce initially at around 5 kPa O_2_ and 0 kPa CO_2_ balanced by N_2_, with film permeability of 400 mL O_2_/m^2^·d·atm.

#### Control experiment

Followed the conventional air packaging (approximately 21 kPa of O_2_) of lettuce. The bags were sealed and then some perforation was made into the bags to keep atmospheric conditions.

### Analysis of headspace inside the packages during storage

In each experimental sampling day, the concentrations of O_2_ and CO_2_ were recorded in 6 packages. A gas analyzer was used to perform all of the measurements as demonstrated by Jacxsens et al. (1999).

### Electrolyte leakage

Electrolyte leakage was recorded just immediately after the processing of fresh-cut lettuce and every consecutive day of following up other parameters. The purpose was to conclude the probable tissue weakening according to López-Gálvez et al. (2015).

### Microbial analysis

The growth of mesophilic bacteria and yeasts was measured as demonstrated by López-Gálvez et al. (2015).

### Sensory quality

Six expert researchers in the field of fresh-cut packaging assessment were investigated the sensory evaluation. A hedonic scale from 1–9 (1 = lowest quality score, 5 = limit of acceptance and, 9 = highest quality score) was used to evaluate the overall sensory quality for fresh-cut lettuce. Browning, off-odors development and dehydration condition were also evaluated on a 1–5 scale (1 = highest quality score, 3 = limit of acceptance and 5 = poorest quality value). The decision of acceptation or rejection of fresh-cut lettuce packages during storage was fully be determined by the expert panelist.

### Extraction and analysis of volatiles

The extraction and analysis of volatile were directed as demonstrated in López-Gálvez et al. (2015).

### Identification and quantification of volatiles

For identification and quantification of volatiles electron impact mass spectra (EI-MS) was scrutinized by Chemstation (Agilent Technologies, USA). For comparison and identification, NIST-database was used ideal. A relative calibration procedure (known concentrations of standards) was used for quantification.

### Statistics and experimental design

The different storage treatments were compared in three independent experiments. During the storage time, six packages were investigated on each sampling day. To compare variations among treatments ANOVA and Brown–Forsythe tests were used. SPSS 19.0 software was used for Tukey or Dunnett T3 tests (*p* ≤ 0.05) to perceive among the treatments.

## Results and discussion

### Gas concentration throughout storing

**Fig. 1:**
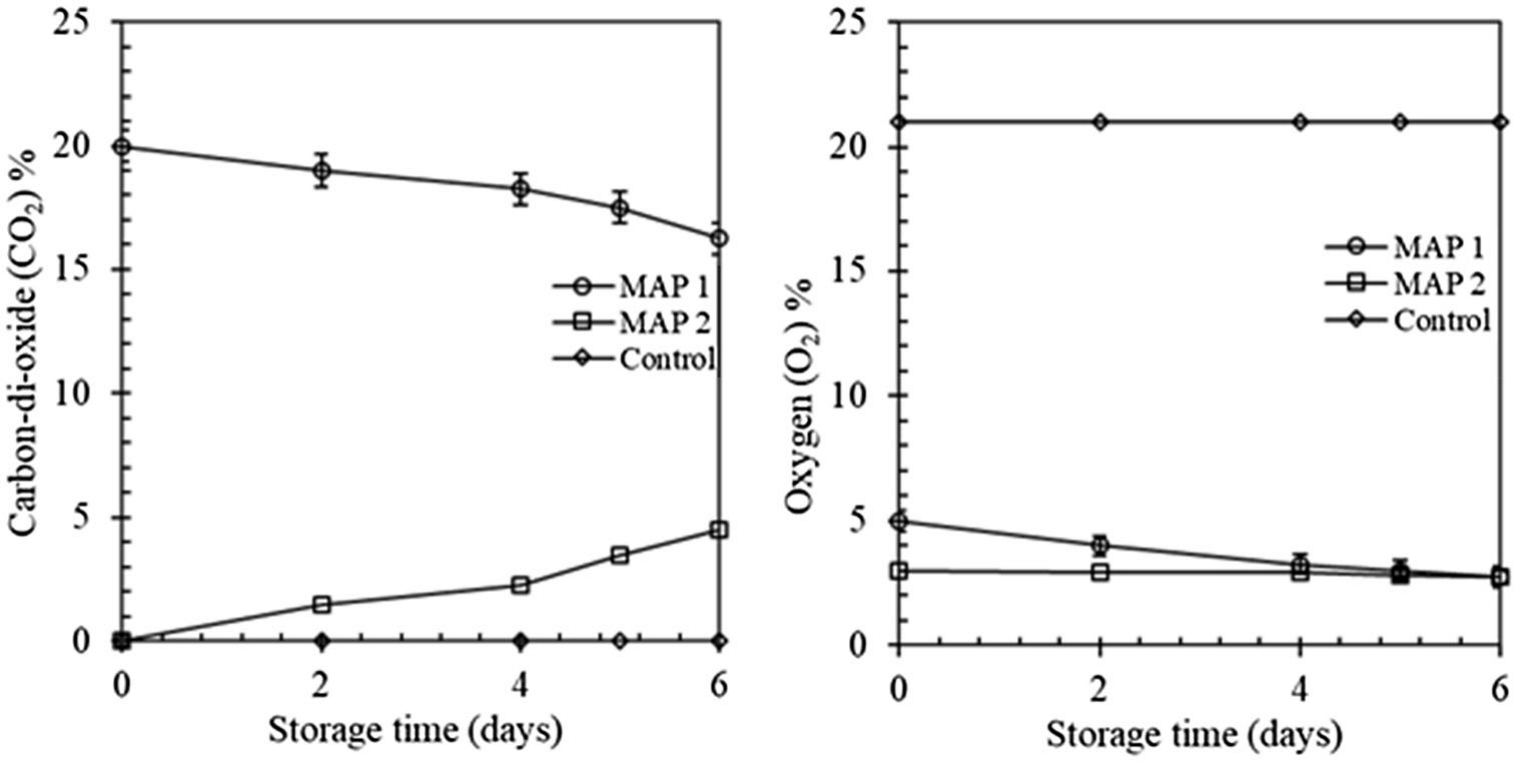
CO_2_ and O_2_ concentration inside packages of lettuce samples throughout storing time. Values are mean ± SD of 6 samples from each treatment

The concentrations of gas inside the packages of fresh-cut lettuce of various treatments is showed in Fig. 1. The level of O_2_ significantly diminished in both treatments. O_2_ concentration decreased from 5 to 2.5 kPa and 5 to 2.4 kPa on the packages of MAP 1 and MAP 2 treatments, respectively. There was a small accumulation of CO_2_ over time (Fig. 1) in MAP 1, and levels raised up to 21 kPa throughout the storage. This slight increase of CO_2_ might be attributed to the respiration process mainly needed to synthesize molecules for tissue restoration (Mattos et al., 2013).

As expected, the O_2_ concentration was diminished and the CO_2_ concentration was amplified over time due to the respiration sensation of lettuce and penetrability of packaging materials ( Corbo et al., 2010; Ölmez and Akbas, 2009). These differences in O_2_ and CO_2_ concentration inside the MAPs were mainly due to the film used (Escalona et al., 2006; Silveira et al., 2014). Since the film has a low CO_2_ transmission rate, it did not allow CO_2_ to evolve from fresh-cut lettuce to be transmitted out. Jacxsens et al. (1999) reported that the gas evolution is affected by the film’s porousness which is temperature-dependent and can be described by the Arrhenius equation.

### Evaluation of mesophilic bacteria and yeasts

**Fig. 2:**
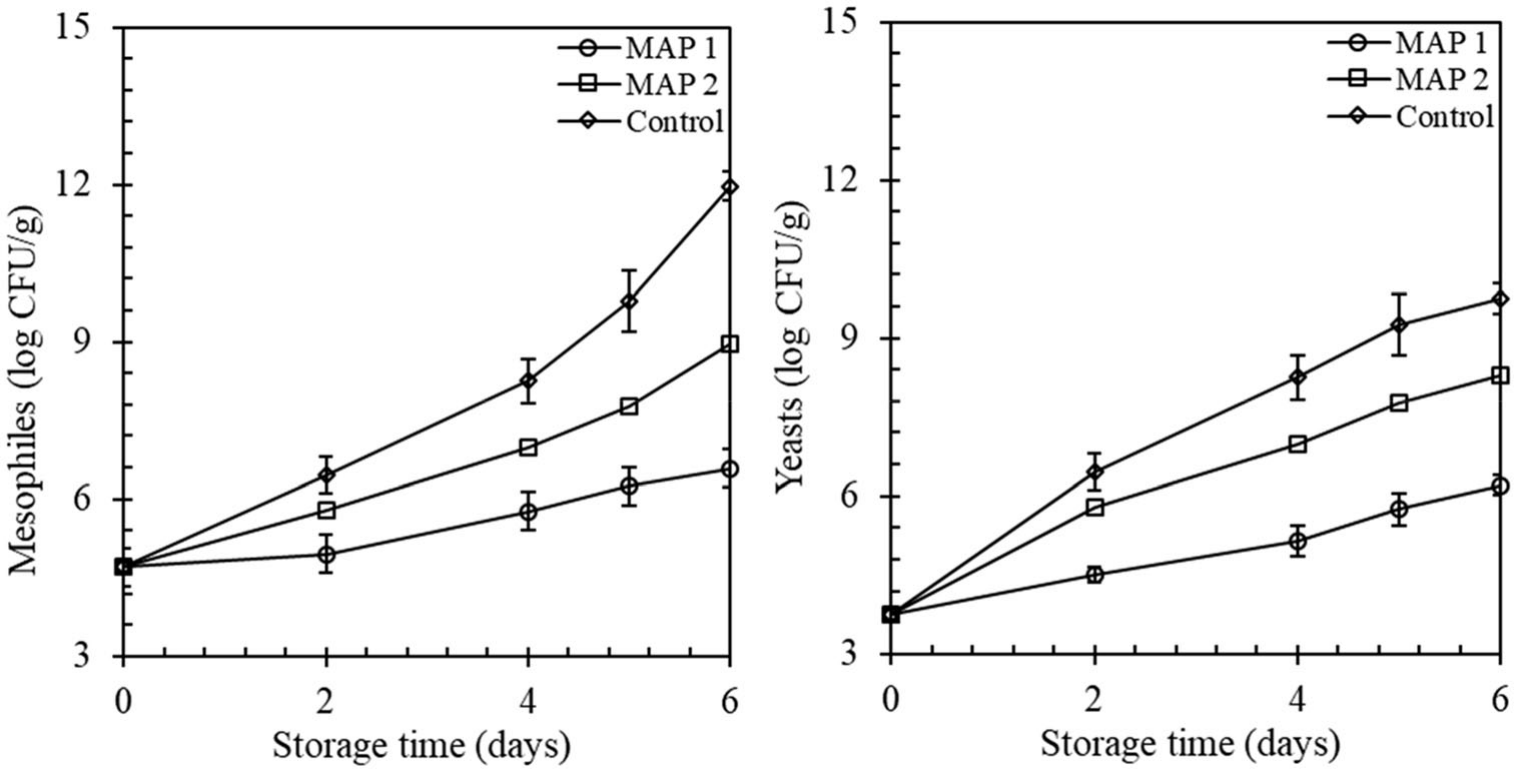
Development of mesophiles and yeasts (log CFU/g) in lettuce samples during the stored period. Values are mean ± SD of 6 samples from each treatment

Mesophilic counts were increased over the time of fresh-cut iceberg lettuce storage in all treatments (Fig. 2). High CO_2_ levels of MAP 1, affected meaningfully the development of mesophilic bacteria (*p* < 0.05) paralleled to low CO_2_ (MAP 2). There were lower mesophilic counts observed in fresh-cut lettuce stored under MAP 1 at days 3, 4, and 5 in both experiments compared to MAP 2. At the beginning of storage, just after washing of lettuce, the mesophilic count was 4.70 ± 0.12 log CFU/g and, at the end of storage (6 days) were 6.58 ± 0.27 log CFU/g and 8.95 ± 0.16 log CFU/g in MAP 1 and MAP 2 respectively. Mesophilic counts were higher in the control experiment as expected. The inhibition of microbial populations in high CO_2_ conditions has been reported in other fresh-cut fruits and vegetables (Allende et al., 2002, Allende et al., 2004; Amanatidou et al., 2000a). So, a higher inhibitory effect on bacterial growth in our experiments might be due to the higher CO_2_ concentration inside the packages. For fresh-cut fruits and vegetables, a targeted criterion of total aerobic psychrotrophic count (TPC) is commonly set at 5 log CFU/g with a maximum acceptable limit of 8 log CFU/g (Jacxsens et al., 2001). In our experiments, the initial TPC was less than 5 log CFU/g irrespective of sample packaging conditions, indicating a good opening biological quality products. Samples stored under control and low level of CO_2_ (MAP 2), mesophilic counts were observed greater than 8 log CFU/g after day 5 (Fig. 2). Samples could not have an extended shelf life except the sample stored at a high CO_2_ atmosphere permitting the TPC criteria.

The development of yeasts is another microbiological spoilage parameter of fresh-cut produce. Yeast counts were investigated over the stored period of fresh-cut lettuce (Fig. 2). In the beginning, the counts were 3.75 ± 0.11 log CFU/g and gradually increased with the storage time. Finally, the values were pushed to 6.21 ± 0.12 log CFU/g and 8.29 ± 0.04 log CFU/g at day 6 in MAP 1 and MAP 2 respectively. A significantly (*p* < 0.05) lower yeasts count was always observed in MAP 1 compared to MAP 2 and control experiments. Statistically significant (*p* < 0.05) transformation was noticed on day 4 and 5.

A similar trend was observed by Jacxsens et al. (1999) and Escalona et al. (2006). The acceptable limit of yeasts recommended of log CFU/g (Uyttendaele et al., 2006) was reached by all treatments at the initial stage. In all experiments, higher growth rates of mesophilic bacteria and yeasts were observed at 7 °C. Our results are comparable with Francis et al. (1999) and Abriouel et al. (2008) who demonstrated that the behavior of microbial growth is affected by temperature, O_2_ and CO_2_ atmospheres.

### Tissue integrity (electrolyte leakage)

**Fig. 3:**
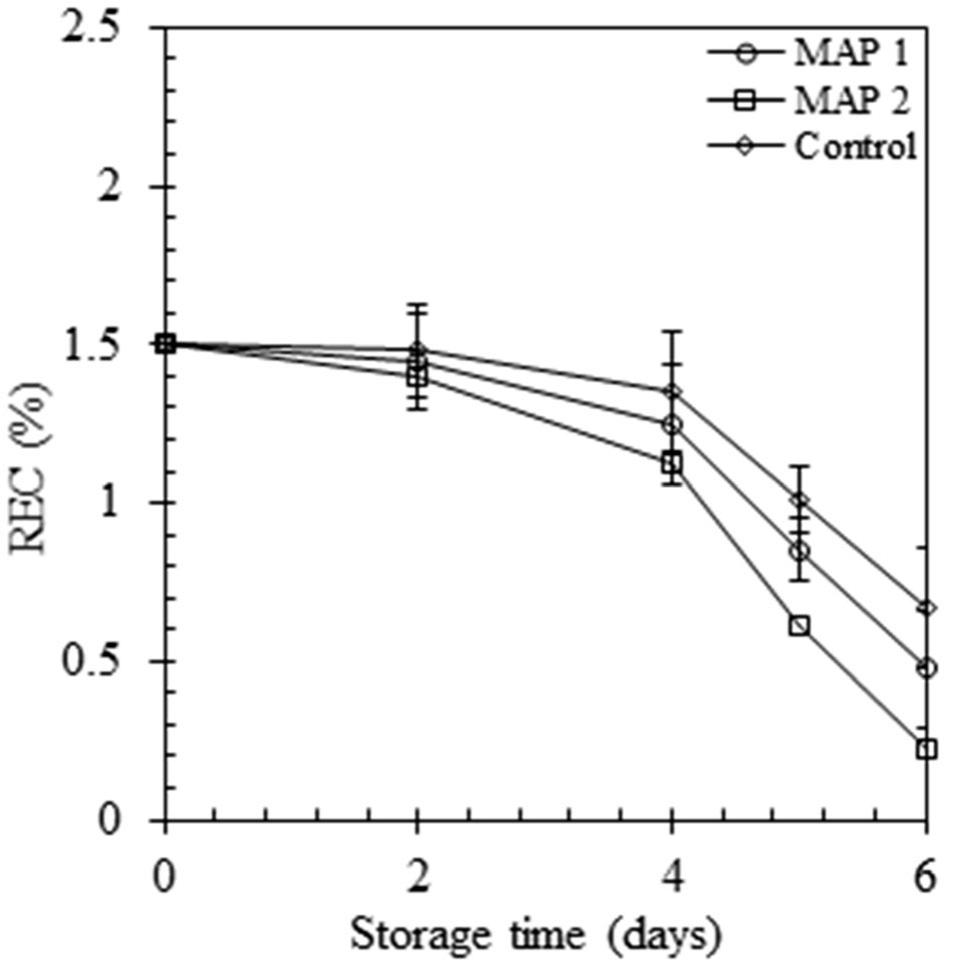
Relative electrolyte leakage of samples. Values are mean ± SD of 6 samples from each treatment

The measurement of electrolyte leakage (EL) is an indirect way of assessing cell membrane damage of fresh-cut lettuce (Bajji et al., 2002). Measurement of tissue integrity is considered a prerequisite approximation for the commercialization of fresh-cut fruits and vegetables (Rolny et al., 2011). The EL values were declined during storage in both treatments and maintained equilibrium from day 2 to the rest of the stored period. Higher tissue electrolyte leakage was observed for the samples stored in high CO_2_ (Fig. 3) atmospheric conditions compared to low CO_2_ atmospheric. The deterioration of membrane integrity by cutting or minimal processing resembles an intensification in EL as a consequence of injury in tissue absorbency and REC (%) falls laterally storing. This was accredited to wound healing of cut tissues and our results were comparable with other investigations (Baert et al., 2009; Fan and Sokorai, 2005).

### Browning in cut-edges and intact surface

**Fig. 4:**
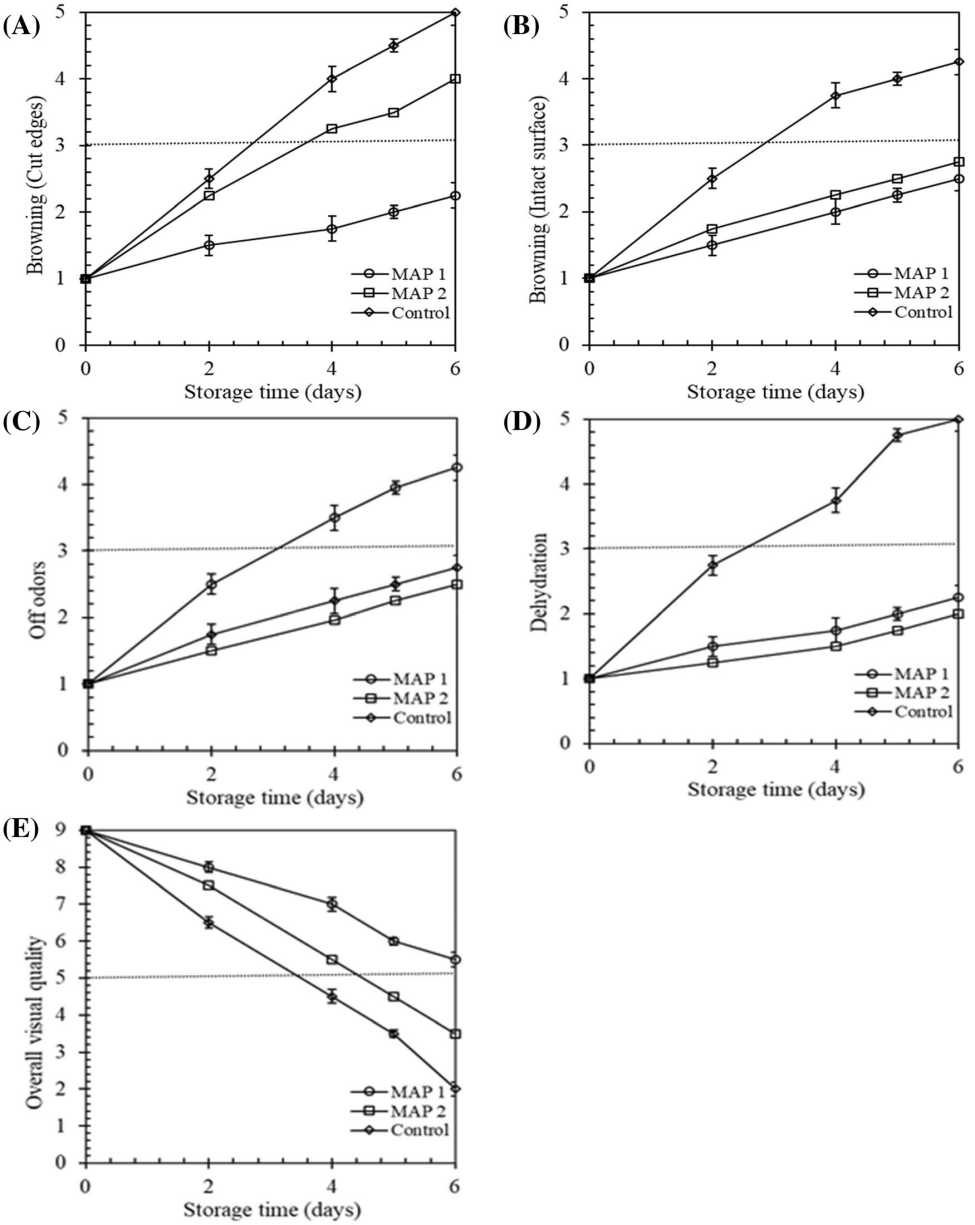
Browning in cut edges (**A**), browning in intact surfaces (**B**), off-odor (**C**), dehydration (**D**) and overall visual quality scores (**E**) of lettuce samples. Values are mean ± SD of 6 samples from each treatment

It´s well known that browning has a noteworthy role in the quality worsening of fresh-cut lettuce. Several studies reported that the browning can be varied among the lettuce types and high CO_2_ atmospheres which is beneficial for the control of browning (López-Gálvez et al., 1996). Cut edge browning is a very imperative parameter for fresh-cut lettuce to mark it incompatible to the consumers (Watkins, 2000). In our study, browning in cut edges and the intact surface was detected clearly and increased during storage and until the end of the experiments (Fig. 4A and B). In both of the experiments browning in cut edges was significantly (*p* < 0.05) greater in fresh-cut lettuce stored under MAP 2 compared to MAP 1. This indicates that high CO_2_ had a significant consequence on retarding cut edge browning of fresh-cut iceberg lettuce. The high CO_2_ atmospheric fresh-cut iceberg lettuce had higher scores and remained accepted until the 5th day of storage from the viewpoint of browning (cut surface and intact surface). Our results was in agreement with the finding of Escalona et al. (2006) who also reported that a CO_2_ level of 15 kPa can retard the browning cut edge and the intact surface of fresh-cut butter-head lettuce. Several studies also demonstrated that an elevated CO_2_ atmosphere packaging reduces the phenolic production and browning reaction (Amanatidou et al., 2000b; Jianglian, 2013; Uddin et al., 2020; Watkins, 2000).

### Off-odors development

Higher intensity of off-odors was developed in fresh-cut lettuce stored under MAP 1 compared to MAP 2 and control (Fig. 4C). The difference was statistically significant (*p* < 0.05). Lettuce preserved under a low CO_2_ atmosphere (MAP 2) always remained acceptable (Off-odors score <3) whereas lettuce stored in MAP 1 exceeded the limit of acceptance after day 3. Our surveillance was strongly correlated with López-Gálvez et al. (1996) and Garcia and Barrett (2002) who reported that accumulation of ethanol and acetaldehyde concentration is responsible for off-odors in diverse fruits stored at minimal O_2_ and/or elevated CO_2_ condition_._

### Development of dehydration

The development of dehydration was not statistically significant (*p* > 0.05) among the treatments throughout the storing time (Fig. 4D) except for the control treatment. Osmotic gradient could be the coherent reason behind dehydration as Ahmed et al. (2016) reported in his work.

### Overall visual quality

**Fig. 5:**
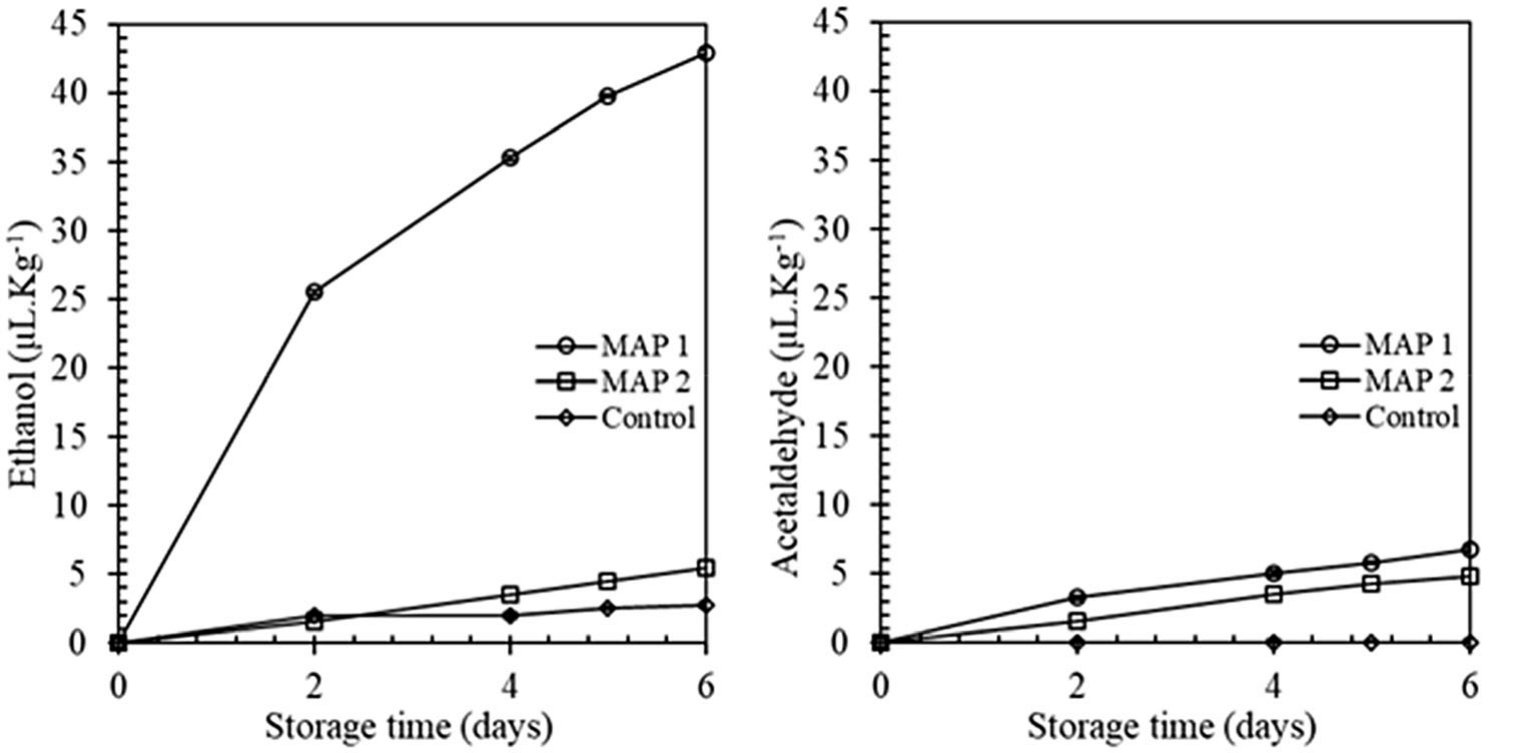
Evolution of ethanol and acetaldehyde (µL/kg) in lettuce samples during the storage period. Symbols are the means of duplicate measurements

Overall visual quality was higher in MAP 1 compared to MAP 2 all replications (Fig. 4E). There were significant differences between MAP 1 and MAP 2 (*p* < 0.05), the scores of overall visual excellence at days 2, 4 and 5 in experiment 1, and at days 4 and 5 in experiment 2. On day 4, the overall visual quality score was below the appropriateness inception (score = 5) for MAP 2, while lettuce stored under MAP 1 was still satisfactory from the visual superiority argument of view at the end of the experiment (Fig. 5).

### Volatile metabolites accumulation analysis

Significantly different levels of ethanol and acetaldehyde accumulation were observed in MAP 1 compared to MAP 2 treated fresh-cut iceberg lettuce. Results specified that the development of severe off-odors was due to a high level of CO_2_. Research investigated that, the off-odors are the sensation of a high level of volatiles (Mishima et al., 2008). Several other studies had similar results (Jianglian, 2013; Lonchamp et al., 2009). Mishima et al. (2008) reported that off-odors development in crisp-head lettuce, when exposed to high CO_2_, was linked with an elevated level of acetaldehyde and ethanol. It was reported high CO_2_ atmospheric storage condition is responsible for the formation of acetaldehyde and ethanol in both intact heads and cut midribs of crisp-head lettuce and other vegetables at 2.5 °C or above (Ahmed et al., 2016; Deza-Durand and Petersen, 2014; Lonchamp et al., 2009).

This research revealed that storage under high CO_2_ retarded the evolution of mesophilic bacteria and yeasts. CO_2_ level of 20 kPa delayed the enzymatic browning of fresh-cut iceberg lettuce and overall visual quality was also within the acceptable limit. The production of off-odors in a high CO_2_ atmosphere was found due to the accumulation of ethanol and acetaldehyde. From the viewpoint of commercialization, neither the high O_2_ nor high CO_2_ condition was recommended to assure a good sensorial and microbial excellence lettuce. Further research will need to be carried out to distinguish the solution to diminish the development of off-odors inside the package.
